# Assessing a Possibility of Divergent Metabolic Responses to Diet Adjustment and Changes of Eating Behaviours in Female Schizophrenia Patients

**DOI:** 10.3390/nu17071198

**Published:** 2025-03-29

**Authors:** Mariola Friedrich, Joanna Sadowska

**Affiliations:** Department of Applied Microbiology and Human Nutrition Physiology, Faculty of Food Sciences and Fisheries, West Pomeranian University of Technology, ul. Papieża Pawła VI 3, 71-459 Szczecin, Poland; mariola.friedrich@zut.edu.pl

**Keywords:** schizophrenia, nutrition, blood parameters, anthropometric parameters, individual responses

## Abstract

**Background/Objectives:** Individuals with schizophrenia are particularly susceptible to overweight, obesity, and metabolic disorders. This study was aimed at assessing the effects of approved diet adjustments, changed nutrition regimes, and eating behaviours on carbohydrate–lipid metabolism. **Methods**: This 3-year study involved 52 residents of a 24 h social welfare home for the chronically mentally ill. Diet adjustment involved balancing the diet energy content and nutrition value as well as changing the sources of basic nutrients. Both metabolic (concentrations of glucose, triglycerides, total cholesterol, and its HDL and LDL fractions) and anthropometric (body weight, waist circumference, hip circumference) parameters as well as body composition were monitored. **Results**: In almost all the subjects, including 12 female schizophrenia patients, diet adjustment and nutrition supervision resulted in beneficial changes in the parameters monitored. The exceptions were three women suffering from schizophrenia, who were sisters, in which glucose concentration declines (5.27 ± 0.22 mmol/L vs. 4.05 ± 0.36 mmol/L) were associated with increased concentrations of triglycerides (0.72 ± 0.17 mmol/L vs. 0.94 ± 0.32 mmol/L), total cholesterol (4.69 ± 0.70 mmol/L vs. 5.44 ± 0.38 mmol/L), and its LDL fraction (2.98 ± 0.65 mmol/L vs. 3.80 ± 0.41 mmol/L), as well as with a decreased HDL cholesterol fraction (1.38 ± 0.04 mmol/L vs. 1.30 ± 0.06 mmol/L). However, the observed changes were not statistically significant. **Conclusions**: It is concluded that diet adjustment and the improvement of nutrition regimes for people with schizophrenia does not always translate into improved parameters of carbohydrate–lipid metabolism.

## 1. Introduction

As shown by numerous studies, individuals diagnosed with schizophrenia are particularly susceptible to being overweight and obese, which results, inter alia, in dysregulation of the neuroendocrine system—the dysregulation being additionally intensified by the antipsychotic medications applied in the treatment of schizophrenia [1,2]. This is associated with a number of metabolic disorders, including insulin resistance, type 2 diabetes, dyslipidaemia, and related cardiovascular diseases and premature deaths [3]. It is therefore extremely important that, during schizophrenia treatment, basic metabolic and anthropometric parameters be monitored and that the patients be provided with information on correct nutrition, body weight control, and physical activity.

Therefore, it was decided to investigate whether a dietary correction, accepted by social welfare home residents, and nutritional supervision would improve selected metabolic and anthropometric indicators of the health status of the studied mentally ill residents, including those with schizophrenia. It was assumed that the improvement of their diet’s composition would be reflected in the improvement of metabolic and anthropometric parameters, despite the medications taken and different metabolic conditions related to the main disease experienced by the resident, as indicated by literature data [1,2,3]. The results of the conducted studies were published in the papers of Friedrich et al. [4,5,6,7]. However, during a detailed individual analysis of the obtained data, attention was drawn to the different results obtained in three patients, who after decoding the questionnaires turned out to be sisters. The aim of this study was to draw attention to the possibility of an individual metabolic response to a correction of diet composition in people with schizophrenia.

## 2. Materials and Methods

This 3-year study involved all 52 residents of a 24 h social welfare home (SWH) for the chronically mentally ill. The study group included 34 men aged 59.2 ± 12.5 (27–80) and 18 women aged 64.0 ± 12.5 (27–80) residing at SWH for at least 4 years. Inclusion criteria: living in a social welfare home where the research was conducted, voluntary consent to the research expressed by the resident or their legal guardian, diagnosed mental illness. Exclusion criteria: lack of consent of the resident or legal guardian, negative attitude of the resident towards cooperation, mental (lack of contact with the resident) or physical (condition preventing participation in anthropometric and biochemical tests) inability to participate in the study. Based on the written consent of the non-incapacitated residents, the legal guardians of the totally incapacitated patients, SWH management, kitchen staff, and the Bioethics Commission of the Regional Medical Chamber in Szczecin (No. 14/KB/V/2013), the patients were provided with comprehensive health-promoting nutrition-focused education; their diets were adjusted and the nutrition regime was supervised. The nutrition-focused education was led by a dietician, assisted by a psychologist, a nurse, and a physiotherapist.

The diet was adjusted by changing the sources of basic nutrients—i.e., proteins, carbohydrates, and lipids—to more highly recommended or health-promoting ones and by balancing its energy and nutritional value appropriately for gender and age, considering the primary illness and comorbid conditions. The amount of red meat and pork sausages in the diet was significantly reduced, replacing them with poultry and fish; full-fat dairy products were replaced with cottage cheese and natural probiotic yoghurts. Products containing purified and processed complex carbohydrates with a high glycemic index were replaced with whole-grain products with a low glycemic index. Fats that are a source of saturated fatty acids, excessive amounts of *n*-6 polyunsaturated fatty acids and trans fatty acids were replaced with fats containing monounsaturated and *n*-3 polyunsaturated acids. The amount of choline sources, vegetables (including naturally fermented vegetables), and seasonal fruits and herbs in the diet was increased. Highly processed products were eliminated from the diet and the amount of kitchen salt and sucrose added to dishes was limited. The technology of preparing dishes was changed to a more health-promoting one (frying was replaced with stewing or baking). A detailed description of the changes is to be found in papers published by Friedrich et al. [4,5]. All those interventions were meant for the SWH residents to fully accept in their nutrition regime (a changed diet composition and altered meal preparation techniques), eating-related behaviours (stopping food throw-outs and stealing food from other residents, etc.), and the structure of residents’ own food purchases. The assessment involved monitoring basic metabolic parameters: concentrations of glucose, total cholesterol (TC), LDL I HDL cholesterol fractions (LDL-C, HDL-C), and triacylglycerols (TG), as well as anthropometric parameters: body weight, body mass index (BMI), waist circumference (WC), hip circumference (HC), waist to hip ratio (WHR), and weight to height ratio (WHtR). Blood for biochemical tests was collected after fasting, between 7:30 and 8:00 a.m., from the basilic vein, by the nursing team from the SWH. Analyses were performed in the Central Laboratory of the District Hospital in Szczecin. The tests were performed in a closed system, using Roche Diagnostica reagents on a COBAS C6000 device (Roche Diagnostics International AG, Rotkreuz, Switzerland). Taking into account the blood lipid parameters, the plasma atherogenic ratios were calculated as follows:

TC [mmol/L]/HDL-C [mmol/L]–Castelli’s Risk Index I (CRI-I) [8];

LDL-C [mmol/L]/HDL-C [mmol/L]–Castelli’s Risk Index II (CRI-II) [8];

TG [mg/dL]/HDL-C [mg/dL] [9].

Anthropometric measurements were performed using the classic Martin technique, in the morning. Body height was measured with a SECA 215 stadiometer (accurate to 0.1 cm); waist and hip circumferences were measured using a Gulick anthropometric tape (accurate to 1 mm); body weight was determined using a RADWAG WPT 200.0 medical scale (accurate to 0.1 kg). The BMI (Body Mass Index) was calculated using the formula: BMI = body weight (kg)/height (m)^2^; the WHR (Waist to Hip Ratio) was calculated using the formula WHR = waist circumference (cm)/hip circumference (cm); the WHtR (Waist to Height Ratio) was calculated using the formula WHtR = waist circumference (cm)/height (cm). On the same day, a body composition test was also performed. The measurement was performed using the non-invasive bioimpedance method BIA (Bioelectrical Impedance Analysis) using the Bodystat 1500MDD device from Bodystat Ltd. (County Down, UK). Anthropometric and biochemical analyses were performed twice—before and after the completion of the nutritional intervention. The detailed methodology and the results for the whole group were described in Nutrients [4] and in Psychiatria Polska [6].

Most of the individuals monitored showed larger or smaller beneficial changes; however, 3 female schizophrenia patients, sisters aged 59, 62, and 64, responded differently to diet adjustment and the changed nutrition regime compared to the remaining women with schizophrenia (*n* = 12), aged 61.7 ± 10.9. While the nutrition regimes as well as food purchases were the same in all the patients [6], it was the carbohydrate–lipid metabolism data of the three females that alerted the present authors to a divergent response.

For the results obtained from 12 women, the normal distribution was confirmed by the Shapiro–Wilk test, homogeneity of variance was confirmed by Levene’s test, and significance was tested using Student’s *t*-test for paired samples (*p* ≤ 0.05). For the results obtained in the group of 3 women–due to the lack of possibility of confirming the homogeneity of variance and normality of distribution due to the small size of the group, statistical analysis was performed using the nonparametric Wilcoxon signed-rank test at *p* ≤ 0.05. The Statistica^®^ 12.0 software program was used (TIBCO, Palo Alto, CA USA).

During the period of study, the three females were treated with risperidone and received no additional medication. The remaining 12 female schizophrenia patients were treated with olanzapine (3 individuals), clozapine (5), olanzapine + clozapine (1), haloperidol (1), quetiapine (1), and perazine (1). Additional medications most frequently involved anti-hypertension (Tisercin, Conor 5, Avedol) and anti-anxiety and stress-reducing drugs (Lorafen, Relanium, Hydroxyzine).

## 3. Results

Table 1 presents the results of biochemical blood parameters in the field of carbohydrate and lipid metabolism in the three sisters studied. After the nutritional intervention, a decrease in glucose concentration was observed (5.27 ± 0.22 mmol/L vs. 4.05 ± 0.36 mmol/L), as well as an increase in TG (0.72 ± 0.17 mmol/L vs. 0.94 ± 0.32 mmol/L), TC (4.69 ± 0.70 mmol/L vs. 5.44 ± 0.38 mmol/L) and LDL-C concentrations (2.98 ± 0.65 mmol/L vs. 3.80 ± 0.41 mmol/L), which was accompanied by a decrease in HDL-C concentrations (1.38 ± 0.04 mmol/L vs. 1.30 ± 0.06 mmol/L). However, the observed changes were not statistically significant. The results for the remaining 12 women in this regard were presented in a publication by Friedrich et al. [4].

As a consequence, the cardiovascular disease risk indicators were observed to increase in the three sisters examined (however statistically insignificant), whereas they dropped down in the other 12 women, being statistically significant for the indicator TC/HDL-C (4.93 ± 1.66 vs. 4.14 ± 1.52, *p* ≤ 0.05) (Table 2).

In the case discussed, in view of the adverse changes in metabolic blood parameters, it was interesting to examine changes in the anthropometric parameters and body composition. With small changes in body weight and BMI, all three women showed a reduced WC (by 1–8 cm) (Table 3).

Similarly beneficial were changes in body composition, which were observed in two women in whom the fat tissue loss was accompanied by an increase in both fat-free body weight and water content; the third woman showed an increase in fat content and a decline in her fat-free body weight and water content. The body composition in the remaining 12 women did not change (Table 4). It should be mentioned, however, that—although the fat content in the three women declined by an average of 2.3% (the decline in the remaining schizophrenia patients amounting to 0.2%)—the change was largely due to the loss of fat on the hips, the circumference of which declined by an average of 7 cm (the average in the remaining women amounting to 3.7 cm [7].

## 4. Discussion

Schizophrenia and the medications associated with its treatment affect the body’s metabolism and can be regarded as a high-risk cardiovascular disease factor. This was why the decreased glucose concentration, resulting from diet adjustment, was so important in this study; in 12 female residents with schizophrenia, it was accompanied by reduced concentrations of TG, TC, and LDL-C as well as an increased HDL–C concentration [4]. On the other hand, the decreased glucose concentration in the three female patients discussed in this paper was accompanied by increased concentrations of TG (initially extremely low), TC, and LDL-C, accompanied by a reduced HDL-C concentration. As a result, it was found that the risk indicators of cardiovascular diseases increased in three of the studied sisters, while they decreased in the remaining 12 women. The values recorded in the three women prior to their diet adjustment were within the admissible limits for females, whereas the adjustment was followed by increased values that were either close to the allowable limits or exceeded them. This points to a risk of sclerosis and metabolic syndrome, particularly in view of the high BMI, WC values far beyond 80 cm, and body fat tissue content found in the three women.

Numerous studies evidence that carbohydrate–lipid metabolism disorders are more than twice as frequent in people with schizophrenia as in the general population [10]. It has also been surmised that schizophrenia and cardiovascular diseases can have joint genetic underpinnings [11]. In addition, some antipsychotic drugs, particularly clozapine and olanzapine, act in favour of carbohydrate–lipid metabolism disorders and type 2 diabetes [1,12]. It has been suggested that the drugs act by, inter alia, inhibiting glucose metabolism and transport to cells, thus contributing to the emergence of insulin resistance and its consequences [13]. The three women were treated with risperidone, which typically causes hyperprolactinemia [14] and weight gain [15], but not severe metabolic disturbances.

Saturated fatty acids (FAs), by affecting the cell wall structure and insulin receptor activity, act in favour of the initiation of glucose metabolism disorders and are potentially responsible for insulin resistance. The diet adjustment involved, inter alia, the elimination of saturated FAs and the replacement of them with polyunsaturated FAs (from olive and rapeseed oils, fatty fish). Hence, the observed decline in the blood glucose concentration in the women examined could have resulted from their reduced consumption of saturated FAs after diet adjustment, and thus from improved cell insulin sensitivity [16]. However, also important in the glucose concentration decline must have been the lowered consumption of simple carbohydrates, reduced dietary glycaemic load, the increased consumption of components involved in correct carbohydrate–lipid metabolism, and the regular meal schedule (2.5 h between-meal intervals) [4]. Why then did the pattern of subsequent metabolism stages, those associated with glucose concentration decline, differ in the three women from that shown by the remaining 12?

Generally, medium- and long-chain FAs serve as ligands of the GPR120 receptor [16,17]; however, recent research shows that FAs are metabolised differently in patients with schizophrenia [18]. The blood serum FA concentration in these patients has been found to be disassociated from the GPR120 receptor, which is suggested to result from GPR120 insensitivity. The literature shows that GPR120 protein synthesis depends on both genetic and environmental factors; GPR120 gene polymorphisms may hamper the synthesis of the protein. This effect was observed by Vestmar et al. [19] in the Danish population they studied. They concluded that the R27OH variant of the GPR120 gene was associated with the GPR120 receptor expression being lower by 70%, compared to individuals free of the mutation.

Is it then possible that a change in the GPR120 receptor polymorphism in the three women resulted in the weakening of its function to a much higher degree than in the remaining women with schizophrenia? That such a mechanism could be involved might be indicated by the fact that GPR120 inhibits lipolysis [20], which was found to be quite intensive in the sisters examined, as indicated by the changes in their WC, HC, and body composition. However, what causes endogenous FAs to be used to change the biosynthesis of lipoproteins? There is no evidence at present that GRP120 directly affects the blood lipid profile. However, as the anti-inflammatory properties of the receptor protein, confirmed in numerous studies [17], can be key in its indirect influence on atherogenesis risk reduction, the decline in its sensitivity might suggest an opposite direction of changes. The distribution and metabolism of lipids depends on numerous factors, including diet composition and the dietary contents of GPR120 agonists; this factor, however, was comparable in the entire group of patients, which resulted from their 24 h residence in the SWH, an identical diet adjustment, and nutritional supervision [7].

On the other hand, considering the possibility that changes in the GPR120 receptor polymorphism of the three women resulted in the receptor’s function weakening being stronger than that in the remaining patients with schizophrenia, it seems plausible that the glucose concentration decline they showed could have been caused by diet adjustment rather than by the involvement of the GPR120 receptor protein. The results of studies on the protein’s role in carbohydrate metabolism are ambiguous; although, one study has shown that the genotype associated with the receptor does not affect tissue insulin sensitivity, insulin resistance, or glucose tolerance [21], other studies have shown a linear reverse correlation between the blood content of the protein and HOMA-IR [22]. Therefore, the GPR120 receptor is regarded as playing a significant part in carbohydrate metabolism regulation by affecting cellular glucose uptake and the function of pancreatic β cells [23].

In their long-term experience with diet adjustments and their beneficial effects on carbohydrate–lipid metabolism, the present authors only once encountered a response different than expected. This happened in some women afflicted with mammary cancer who, compared to other similarly afflicted patients receiving identical diet adjustments, with a statistically significant glucose concentration decline showed increased concentrations of TG, TC, and LDL-C, a reduction in HDL-C, and an increased estradiol concentration [24]. Considering that mammary cancer is classified as a genetic condition as well as with hormone-and diet-dependent tumours, attention was then drawn to this effect, which was considered very unfavourable. Although current studies on the role of GPR120 in cancer are at their initial stage only, the positive correlation between fat consumption and the development of some cancer forms [25]—including those that are genetically linked as well as those that are hormone- and diet-dependent—could be related to the contribution of the receptor protein discussed to disturbed carbohydrate–lipid metabolism in some patients.

Schizophrenia is a chronic, serious condition that is difficult to treat. It is also associated with a number of metabolic disorders, intensified by the antipsychotic drugs used in its treatment, and frequently associated with obesity. Therefore, in addition to pharmacological treatment, an efficacious therapeutic intervention in schizophrenia should have a dietary component. A proper diet composition, in addition to its basic function, may also reduce or weaken symptoms of, inter alia, anxiety, lowered mood, stimulation, or aggression [7]. What is equally important, particularly for women, is that a proper diet composition may prevent excessive fat accumulation. In the case of the three sisters with schizophrenia discussed, diet adjustment failed to bring about positive results; these were quite opposite. This shows that it is not always the patient’s missing dietary discipline that is to be blamed for the absence of desired effects. During walk-in therapy, when there is no possibility of monitoring the patient’s dietary habits, the lack of beneficial metabolic changes may raise the doctor’s doubts as to the patient’s credibility, and at the same time the patient may be discouraged from adhering to a rational diet and experience various negative emotions.

## 5. Conclusions

An analysis of the results obtained allowed us to conclude that diet adjustment in schizophrenia can yield positive metabolic outcomes, but that individual responses may vary due to underlying genetic and physiological factors.

## Figures and Tables

**Table 1:** Impact of diet correction on selected parameters of carbohydrate–lipid metabolism, SWH residents suffering from schizophrenia—the case of the three sisters; individually, $\bar{x}$ ± SD.

Trait	Women, *n* = 3	
	Before	After
Glucose (mmol/L)	5.49 5.27 5.05 5.27 ± 0.22	4.11 3.66 4.38 4.05 ± 0.36
TG (mmol/L)	0.61 0.63 0.91 0.72 ± 0.17	0.68 0.84 1.30 0.94 ± 0.32
TC (mmol/L)	4.3 4.27 5.49 4.69 ± 0.70	5.10 5.36 5.83 5.44 ± 0.38
HDL-C (mmol/L)	1.42 1.36 1.34 1.38 ± 0.04	1.27 1.25 1.36 1.30 ± 0.06
LDL-C (mmol/L)	2.59 2.61 3.73 2.98 ± 0.65	3.39 3.78 4.22 3.80 ± 0.41

TG: triacylglycerols, TC: total cholesterol, HDL-C: high-density lipoprotein cholesterol fraction, LDL-C: low-density lipoprotein cholesterol fraction.

**Table 2:** Impact of diet correction on the values of indicators determining the risk of cardiovascular diseases, SWH residents suffering from schizophrenia, the case of the three sisters vs. other women with schizophrenia; individually for *n* = 3, $\bar{x}$ ± SD.

Trait	Women, *n* = 12		Women, *n* = 3	
	Before	After	Before	After
TC/HDL-C	4.93 ± 1.66 ^b^	4.14 ± 1.52 ^a^	3.03 3.13 4.09 3.41 ± 0.59	4.0 4.29 4.30 4.19 ± 0.17
LDL-C/HDL-C	3.25 ± 1.14	2.81 ± 0.79	1.82 1.92 2.79 2.17 ± 0.53	2.66 3.02 3.10 2.93 ± 0.23
TG/HDL-C	4.24 ± 2.21	3.24 ± 2.05	0.98 1.06 1.56 1.20 ± 0.31	1.22 1.53 2.19 1.64 ± 0.49

TG: triacylglycerols, TC: total cholesterol, HDL-C: high-density lipoprotein cholesterol fraction, LDL-C: low-density lipoprotein cholesterol fraction; ^a,b^—Means marked with different letters in the same line are statistically different, *p* ≤ 0.05.

**Table 3:** Impact of diet correction on selected anthropometric parameters of nutritional status, SWH residents suffering from schizophrenia—the case of the three sisters; individually, $\bar{x}$ ± SD.

Trait	Women, *n* = 3	
	Before	After
Body weight (kg)	81.0 67.0 83.0 77.0 ± 8.72	80.5 70.5 82.4 76.8 ± 8.61
BMI (kg/m^2^)	29.8 24.3 31.4 28.5 ± 3.72	29.6 25.6 31.2 28.8 ± 2.88
WC (cm)	100 89 110 99.7 ± 10.5	92 91 109 97.3 ± 10.1
HC (cm)	112 99 112 107.7 ± 7.51	106 98 102 102.0 ± 4.0
WHR	0.89 0.90 0.98 0.92 ± 0.05	0.87 0.93 1.07 0.96 ± 0.10
WHtR	0.61 0.54 0.68 0.61 ± 0.07	0.56 0.55 0.67 0.59 ± 0.07

BMI: Body Mass Index, WC: waist circumference, HC: hip circumference, WHR: waist-to-hip ratio, WHtR: waist-to-height ratio.

**Table 4:** Impact of diet correction on selected parameters of body composition, SWH residents suffering from schizophrenia, the case of the three sisters vs. other women with schizophrenia; individually for *n* = 3, $\bar{x}\;$ ± SD.

Trait	Women, *n* = 12		Women, *n* = 3	
	Before	After	Before	After
Total body fat (%)	47.2 ± 12.1	47.0 ± 10.2	63.5 62.3 55.7 60.5 ± 4.2	58.3 57.8 58.4 58.2 ± 0.32
Lean body mass (%)	52.7 ± 12.0	53.0 ± 10.2	36.5 37.7 44.3 39.5 ± 4.2	41.7 42.2 41.6 41.8 ± 0.32
Total body water (%)	47.9 ± 6.5	48.1 ± 4.5	43.5 45.2 47.8 45.5 ± 2.17	46.5 48.4 46.2 47.0 ± 1.19

## Data Availability

The data presented in this study are available on request from the corresponding author.

## Abbreviations

The following abbreviations are used in this manuscript:

SWH	Social Welfare Home
CRI-I	Castelli’s Risk Index I
CRI-II	Castelli’s Risk Index II
TG	Triacylglycerols
TC	Total cholesterol
HDL-C	High-density lipoprotein cholesterol fraction
LDL-C	Low-density lipoprotein cholesterol fraction
BMI	Body Mass Index
WC	Waist circumference
HC	Hip circumference
WHR	Waist-to-hip ratio
WHtR	Waist-to-height ratio
FA	Fatty acids
GPR120	G protein-coupled receptor 120
R27OH	Variant of the GPR120 gene
HOMA-IR	Homeostatic Model Assessment
